# Relations between Dentist and Patient

**Published:** 1899-05

**Authors:** 


### SELECTIONS.

### Relations Between Dentist and Patient.

There is one factor in dental practice—as well as in many other affairs of life—which exercises a profound influence on the success or otherwise of the practitioner ; this factor is a knowledge of human nature. Carlyle, in an oft-quoted passage, has stigmatized the dwellers in these islands as "mostly fools," and we all freely admit that we all have some weak point ; perhaps even the cynical philosopher himself had. If we study the weakness of our fellow creatures in order to prey upon them, to deceive them, or to work upon their vanity or their ignorance, we lower our moral nature, and do ourselves infinitely more harm than we do them. But if we take the word of Pope that "the noblest study of mankind is man," if we study human nature with the idea of benefiting by that which is good, and of being helpful to that which is weak, ignorant or misguided, our knowledge of human nature will be a help to ourselves and cannot fail to bring us the respect of those with whom we come in contact, whether patients or not.

And we may here briefly discuss how far it is advisable to make friends of our patients or they of us. We have heard it said, " I do not object to my friend becoming my patient, but I object to my patient becoming my friend." We are inclined to agree with the axiom, though not altogether. There can be no objection to our friend becoming our patient, though it is quite possible to imagine a case in which we had rather it did not occur. But as regards the other clause, we think there can be very little difference of opinion. It is better for the friendly relations between dentist and client to be confined to the consulting room. The fact of a professional acquaintance existing, should give neither party the right to presume upon it in any way. If either chooses to ignore the other's presence under other circumstances he has a perfect right to do so. The unwritten laws of good taste and right feeling will guide those who possess them ; those who do not are not worth worrying about.

It is interesting to watch the change of feeling which has gradually come over the public mind during the years gone by as regards the status granted to various professions. At one time a man, to be a gentleman, was either a fighter or a cleric. Might was right in this life, so the fighter was an important person—and still is. The importance of the cleric lay in his assumed power over the future life—this also lingers to a large extent. In course of time, as right began to assert itself, the lawyer became more important, was admitted to some of the highest positions in the State, and it became recognized that the law, too, was a profession which a gentleman might enter. Medicine, which meekly limped behind her prouder sisters, has raised her head during this century in a remarkable manner, and we have had the satisfaction of seeing ere its close, a medical man as a minister of the crown and another a peer of the realm. If we read the description of medica men and medical students as portrayed in the novels of fifty years ago, we are struck by the difference existing to-day. Dr. Smollet, when serving as "surgeon's mate" on board a man-of-war a hundred years ago, never dreamt of sitting down to the same table as his captain, who was most probably ignorant of his existence on board his vessel. Our specialty has also made great strides of late years, and our position as educated men and useful members of society is freely granted by all right-thinking people. But we are all brought in contact with people who do not only not think rightly, but scarcely seem to think at all. It is when feeling a want of consideration—to say nothing of gratitude—from people of this sort, that our knowledge of human nature helps us, as we know the genus "snob" to be as much a branch of the great human family as it was when Thackeray wrote. Finally, a few words of the great philosopher, Marcus Aurelius, may well be born in mind: "Be satisfied with your business, and learn to love what you were bred to; and as to the remainder of your life, be entirely resigned, and let the gods do their pleasure with your body and your soul. And when this is done, be neither slave nor tyrant to anybody."—*British Journal of Dental Science.*

